# Supplementary material for: The association between polypharmacy and health-related quality of life among non-dialysis chronic kidney disease patients
Source: PLoS One. 2023 Nov 13;18(11):e0293912. doi: 10.1371/journal.pone.0293912 (PMC10642842; doi:10.1371/journal.pone.0293912)
Supplement: S1 Checklist — (DOCX) [file pone.0293912.s001.docx]

STROBE Statement—checklist of items that should be included in reports of observational studies

|  | Item No. | Recommendation | Page  No. | Relevant text from manuscript |
| --- | --- | --- | --- | --- |
| **Title and abstract** | 1 | (*a*) Indicate the study’s design with a commonly used term in the title or the abstract | 2 | This cross-sectional study utilized data from the Medical Expenditure Panel Survey (MEPS) from 2010 through 2019. |
|  |  | (*b*) Provide in the abstract an informative and balanced summary of what was done and what was found | 2 – 3 | We applied multivariate ordinary least squares regression to assess the association between polypharmacy and HRQoL in non-dialysis CKD patients.  Our study found that both major and hyperpolypharmacy were associated with lower HRQoL among non-dialysis CKD patients. |
| Introduction | | | |  |
| Background/rationale | 2 | Explain the scientific background and rationale for the investigation being reported | 4 | The impact of HRQoL on ESRD patients, who mostly require dialysis has been well documented [20–25]. In contrast, only a few studies have evaluated the association of HRQoL and non-dialysis CKD patients. To date, only one study on dialysis patients from a Dutch dialysis center has investigated the impact of polypharmacy on the HRQoL in CKD patients. Dialysis patients had lower PCS scores when the medication threshold was greater than or equal to five and lower MCS scores when the number of medications was between 14 – 27 [26]. More studies are needed in this area to further elucidate this association. To the best of our knowledge, no studies have investigated the impact of polypharmacy on the HRQoL in non-dialysis CKD patients. |
| Objectives | 3 | State specific objectives, including any prespecified hypotheses | 4 | This study aims to determine the association between polypharmacy and HRQol among non-dialysis CKD patients. We hypothesize that higher polypharmacy will be associated with lower HRQoL. In addition, we aim to identify the top 5 prescribed medication classes consumed by non-dialysis CKD patients and examine their prevalence across age groups, sex, levels of polypharmacy, and HRQoL categories. |
| Methods | | | |  |
| Study design | 4 | Present key elements of study design early in the paper | 5 | This was a cross-sectional study conducted using medical expenditure panel survey (MEPS) data from 2010 – 2019 to determine the association between polypharmacy and HRQoL among non-dialysis CKD patients. |
| Setting | 5 | Describe the setting, locations, and relevant dates, including periods of recruitment, exposure, follow-up, and data collection | 5 | This was a cross-sectional study conducted using medical expenditure panel survey (MEPS) data from 2010 – 2019 to determine the association between polypharmacy and HRQoL among non-dialysis CKD patients. |
| Participants | 6 | (*a*) *Cohort study*—Give the eligibility criteria, and the sources and methods of selection of participants. Describe methods of follow-up  *Case-control study*—Give the eligibility criteria, and the sources and methods of case ascertainment and control selection. Give the rationale for the choice of cases and controls  *Cross-sectional study*—Give the eligibility criteria, and the sources and methods of selection of participants | 6 | Study Population  The study population included all non-dialysis CKD patients 18 years and older, living in the US and alive during the data collection period. We selected individuals with CKD using ICD-10 codes N18 (chronic kidney disease) and N19 (unspecified kidney failure) for datasets from 2016 through 2019 and ICD-9 codes 585 (chronic renal failure) and 586 (unspecified renal failure) for datasets from 2010 through 2015.  Exclusion Criteria  As MEPS data is a sub-selection of individuals who participated in the previous year’s NHIS, MEPS collects data from every individual in the target area. It is possible for the status of some of these individuals to have changed (for example, to an institutionalized population since participating in the NHIS). If a change in status occurs, MEPS assigns negative weights, PCS, and MCS scores. Hence, we excluded all participants with a negative weight on the self-administered questionnaire (SAQ). The SAQ is a paper-and-pencil questionnaire that asks questions about health status. We also excluded patients deemed ineligible to participate in the SAQ questionnaire based on the criteria from MEPS or if they had a negative PCS or MSC score. We excluded patients if they were < 18 years old, institutionalized, or had no information available for the specific round. Furthermore, we excluded patients if they had any cancer, were undergoing dialysis (identified from the medical condition files as patients depending on any machine or undergoing hemodialysis), or if MEPS had no medications listed to determine polypharmacy (Fig 1). |
|  |  | (*b*) *Cohort study*—For matched studies, give matching criteria and number of exposed and unexposed  *Case-control study*—For matched studies, give matching criteria and the number of controls per case |  |  |
| Variables | 7 | Clearly define all outcomes, exposures, predictors, potential confounders, and effect modifiers. Give diagnostic criteria, if applicable | 7 – 8 | The dependent variables used in this study were HRQoL measures. We obtained HRQoL variables from the Veterans Rand 12 (VR-12) item health survey for data from 2017 through 2019 and the short form 12 Version 2 (SF-12v2) for data pulled from 2010 through 2016. The VR-12 and SF-12v2 are self-administered questionnaires that have two components each: PCS and MCS. Each component has six separate items. To make the VR-12 and SF-12v2 compatible, AHRQ applied a bridging algorithm to the MEPS data to align the summary scores (PCS and MCS) from the VR-12 and SF-12v2 questionnaires [27]. The PCS and MCS each have a mean summary score of 50 and a standard deviation of 10 in the US population. Higher scores indicate better physical and mental quality of life. The PCS provides greater weight to questions involving role limitations with physical functioning, physical health, pain, and general health. The MCS provides greater weight to questions involving interference with emotional problems, social functioning, and mental health.  The primary independent variable of interest for this study was polypharmacy. To date, there is no universal definition for polypharmacy. Some studies utilized a threshold of 5 or more concomitant medications over a certain period to define polypharmacy, while others employed a different threshold [28]. In this study, we classified polypharmacy based on the number of unique therapeutic classes of prescribed medications taken concomitantly over a specific round (e.g., 6-month data collection period with each yearly released data files designed by AHRQ such that patients in the current year are independent of patients in the previous year with each patient assigned unique weights, and variance estimation strata). We characterized polypharmacy as follows: minor polypharmacy (≤ 4 classes of medications), major polypharmacy (5-9 classes of medications), and hyperpolypharmacy (≥10 classes of medications) [28].  We included other independent variables in this study as explanatory variables. We included demographic variables such as age group (18 to 44, 45 to 64, and ≥ 65 years), race (Hispanic, White, Black, and other race – Asian, Native Hawaiian/Pacific Islander, and Filipino), and marital status (married, and unmarried). Socioeconomic variables, namely insurance coverage (private, public, and uninsured), education attainment (college and more, < college, and missing = 4), and income level (low, middle, and high), were evaluated. Other variables included are census region (Northeast, Midwest, South, and West), physical activity (moderate to vigorous, no moderate to vigorous, and missing = 22), access to care (yes, no, and missing = 5), and comorbidities/risk factors such as diabetes, hypertension, arthritis and cardiovascular diseases (CVD). |
| Data sources/ measurement | 8* | For each variable of interest, give sources of data and details of methods of assessment (measurement). Describe comparability of assessment methods if there is more than one group | 6 – 7 | We extracted demographic variables, socioeconomic status, HRQoL, and mental health conditions from the HC files for each study year. We obtained information on prescribed medications from the prescription medicine files and data regarding medical conditions reported by respondents from the medical condition files. We consolidated individual date variable files into one file using unique identifiers.  Dependent Variable  The dependent variables used in this study were HRQoL measures. We obtained HRQoL variables from the Veterans Rand 12 (VR-12) item health survey for data from 2017 through 2019 and the short form 12 Version 2 (SF-12v2) for data pulled from 2010 through 2016. The VR-12 and SF-12v2 are self-administered questionnaires that have two components each: PCS and MCS. Each component has six separate items. To make the VR-12 and SF-12v2 compatible, AHRQ applied a bridging algorithm to the MEPS data to align the summary scores (PCS and MCS) from the VR-12 and SF-12v2 questionnaires [27].  Independent Variable  The primary independent variable of interest for this study was polypharmacy. To date, there is no universal definition for polypharmacy. Some studies utilized a threshold of 5 or more concomitant medications over a certain period to define polypharmacy, while others employed a different threshold [28]. In this study, we classified polypharmacy based on the number of unique therapeutic classes of prescribed medications taken concomitantly over a specific round (e.g., 6-month data collection period with each yearly released data files designed by AHRQ such that patients in the current year are independent of patients in the previous year with each patient assigned unique weights, and variance estimation strata). We characterized polypharmacy as follows: minor polypharmacy (≤ 4 classes of medications), major polypharmacy (5-9 classes of medications), and hyperpolypharmacy (≥10 classes of medications) [28]. |
| Bias | 9 | Describe any efforts to address potential sources of bias | 6 | As MEPS data is a sub-selection of individuals who participated in the previous year’s NHIS, MEPS collects data from every individual in the target area. It is possible for the status of some of these individuals to have changed (for example, to an institutionalized population since participating in the NHIS). If a change in status occurs, MEPS assigns negative weights, PCS, and MCS scores. Hence, we excluded all participants with a negative weight on the self-administered questionnaire (SAQ). The SAQ is a paper-and-pencil questionnaire that asks questions about health status. We also excluded patients deemed ineligible to participate in the SAQ questionnaire based on the criteria from MEPS or if they had a negative PCS or MSC score. We excluded patients if they were < 18 years old, institutionalized, or had no information available for the specific round. Furthermore, we excluded patients if they had any cancer, were undergoing dialysis (identified from the medical condition files as patients depending on any machine or undergoing hemodialysis), or if MEPS had no medications listed to determine polypharmacy (Fig 1). |
| Study size | 10 | Explain how the study size was arrived at | 10 | Among 339,883 participants in the MEPS data, 932 had CKD, of which 649 met the inclusion criteria (Fig 1). |

Continued on next page

| Quantitative variables | 11 | Explain how quantitative variables were handled in the analyses. If applicable, describe which groupings were chosen and why | 8 – 9 | We assessed the prevalence of the top 5 commonly used therapeutic classes of medications across age groups, sex, degree of polypharmacy, and HRQoL categories. In this case, we dichotomized HRQoL (low PCS, high PCS, low MCS, and high MCS) using the rank procedure in SAS.  Kessler Index (K6) scale is a validated questionnaire used to determine mental conditions with higher values indicating SMD. A dichotomous cut-point of 13 or more was applied to classify SMD, as described by Kessler et al. [29]. |
| --- | --- | --- | --- | --- |
| Statistical methods | 12 | (*a*) Describe all statistical methods, including those used to control for confounding | 9 | Rao-Scott Chi-square test was applied to the categorical variables to determine significant differences in the population characteristics across degrees of polypharmacy. We utilized unadjusted ordinary least squares regression to evaluate the association between the explanatory variables and HRQoL (PCS and MCS). We performed further analysis using multivariable ordinary least squares regression to investigate the independent association between polypharmacy and HRQoL while controlling for other explanatory variables such as sex, age, race/ethnicity, marital status, income level, prescription drug coverage, health insurance coverage, physical activity, access to care, census region, education, number of comorbidities, psychiatric illnesses, and comorbidities/risk factors. We assessed the prevalence of the top 5 commonly used therapeutic classes of medications across age groups, sex, degree of polypharmacy, and HRQoL categories. In this case, we dichotomized HRQoL (low PCS, high PCS, low MCS, and high MCS) using the rank procedure in SAS. Furthermore, we examined the type of specific medication used for each of the top 5 therapeutic classes of medications.  The explanatory variables used in the adjusted model were assessed for multicollinearity using variance inflation factor (VIF), pairwise correlation coefficients, and tolerance indices (TIs). The presence of multicollinearity can lead to imprecise parameter estimates and standard errors, which in turn can affect the overall accuracy of the model [31,32]. All variables had VIF of less than 5 with tolerance levels greater than 0.2, which implies the absence of multicollinearity [31]. All other inference assumptions of the models were examined to ensure that all requirements were met. Given the complex sampling design of the MEPS data, all statistical analyses were performed utilizing strata, clustering, and SAQ weight in testing for statistical differences with a significant level (alpha) of 0.05. All analyses were conducted using SAS software, version 9.4 (SAS institute. Cary, NC). |
|  |  | (*b*) Describe any methods used to examine subgroups and interactions | 9 | Rao-Scott Chi-square test was applied to the categorical variables to determine significant differences in the population characteristics across degrees of polypharmacy. |
|  |  | (*c*) Explain how missing data were addressed | 9 | Missing data was addressed using pairwise deletion. |
|  |  | (*d*) *Cohort study*—If applicable, explain how loss to follow-up was addressed  *Case-control study*—If applicable, explain how matching of cases and controls was addressed  *Cross-sectional study*—If applicable, describe analytical methods taking account of sampling strategy | 9 | Given the complex sampling design of the MEPS data, all statistical analyses were performed utilizing strata, clustering, and SAQ weight in testing for statistical differences with a significant level (alpha) of 0.05. |
|  |  | (*e*) Describe any sensitivity analyses |  |  |
| Results | | | | |
| Participants | 13* | (a) Report numbers of individuals at each stage of study—eg numbers potentially eligible, examined for eligibility, confirmed eligible, included in the study, completing follow-up, and analysed | 10 | Among 339,883 participants in the MEPS data, 932 had CKD, of which 649 met the inclusion criteria (Fig 1). |
|  |  | (b) Give reasons for non-participation at each stage |  | Fig 1 |
|  |  | (c) Consider use of a flow diagram |  | Fig 1 |
| Descriptive data | 14* | (a) Give characteristics of study participants (eg demographic, clinical, social) and information on exposures and potential confounders | 10 | The mean age and standard deviation of CKD patients was 61.55 ± 13.93. Sex was not significantly different across the levels of polypharmacy. Over two-thirds (76.89%) of participants exhibited major or hyperpolypharmacy. CKD patients were predominantly White (62.89%), followed by Blacks (20.66%), Hispanics (10.32%), and other races (6.14%). The proportion of patients with major (49.20%) and hyperpolypharmacy (84.28%) was more pronounced when the number of comorbidities were ≥ 3. All psychiatric illnesses (i.e., depression, SMD, and anxiety) and comorbidities (i.e., diabetes, hypertension, arthritis, CVD) were significantly different (p-value < 0.001) across all degrees of polypharmacy. The most prevalent comorbidity among patients with CKD was hypertension (84.82%). Across psychiatric illnesses, hyperpolypharmacy occurred more frequently in patients with depression (45.30%), SMD (50.43%), and anxiety (54.07%) (Table 1). |
|  |  | (b) Indicate number of participants with missing data for each variable of interest | 8 | Socioeconomic variables, namely insurance coverage (private, public, and uninsured), education attainment (college and more, < college, and missing = 4), and income level (low, middle, and high), were evaluated. Other variables included are census region (Northeast, Midwest, South, and West), physical activity (moderate to vigorous, no moderate to vigorous, and missing = 22), access to care (yes, no, and missing = 5), and comorbidities/risk factors such as diabetes, hypertension, arthritis and cardiovascular diseases (CVD). |
|  |  | (c) *Cohort study*—Summarise follow-up time (eg, average and total amount) |  |  |
| Outcome data | 15* | *Cohort study*—Report numbers of outcome events or summary measures over time |  |  |
|  |  | *Case-control study—*Report numbers in each exposure category, or summary measures of exposure |  |  |
|  |  | *Cross-sectional study—*Report numbers of outcome events or summary measures | 13 | Patients with hyperpolypharmacy had the lowest mean PCS at 29.62 (95% CI: 27.30 – 31.94), which was followed by major polypharmacy at 34.90 (95% CI: 32.68 – 37.12) and patients with minor polypharmacy had the highest PCS at 41.46 (95% CI: 38.89 – 44.03) with a p-value of <0.0001. |
| Main results | 16 | (*a*) Give unadjusted estimates and, if applicable, confounder-adjusted estimates and their precision (eg, 95% confidence interval). Make clear which confounders were adjusted for and why they were included | 9, 13, 17 | The explanatory variables used in the adjusted model were assessed for multicollinearity using variance inflation factor (VIF), pairwise correlation coefficients, and tolerance indices (TIs). The presence of multicollinearity can lead to imprecise parameter estimates and standard errors, which in turn can affect the overall accuracy of the model [31,32]. All variables had VIF of less than 5 with tolerance levels greater than 0.2, which implies the absence of multicollinearity [31]. All other inference assumptions of the models were examined to ensure that all requirements were met.  We performed further analysis using multivariable ordinary least squares regression to investigate the independent association between polypharmacy and HRQoL while controlling for other explanatory variables such as sex, age, race/ethnicity, marital status, income level, prescription drug coverage, health insurance coverage, physical activity, access to care, census region, education, number of comorbidities, psychiatric illnesses, and comorbidities/risk factors.  Patients with hyperpolypharmacy had the lowest mean PCS at 29.62 (95% CI: 27.30 – 31.94), which was followed by major polypharmacy at 34.90 (95% CI: 32.68 – 37.12) and patients with minor polypharmacy had the highest PCS at 41.46 (95% CI: 38.89 – 44.03) with a p-value of <0.0001. Age group, income level, prescription drug coverage, health insurance, physical activity, access to care, number of comorbidities, psychiatric illnesses, diabetes, and arthritis were all significantly associated with PCS based on a significance level of 0.05. CKD patients with depression and SMD had significantly lower PCS than those without (Table 2).  In the adjusted model, the mean PCS was significantly lower (by 3 points) among CKD patients with major polypharmacy when compared with minor polypharmacy [Beta = -3.12, (95% CI: -3.62, -2.66), p-value <0.001]. Similarly, the PCS was four points lower among CKD patients with hyperpolypharmacy compared to those with minor polypharmacy [Beta = -4.56, (95% CI: -5.11, -4.01), p-value <0.001]. |
|  |  | (*b*) Report category boundaries when continuous variables were categorized | 8 – 9 | Kessler Index (K6) scale is a validated questionnaire used to determine mental conditions with higher values indicating SMD. A dichotomous cut-point of 13 or more was applied to classify SMD, as described by Kessler et al. [29].  We assessed the prevalence of the top 5 commonly used therapeutic classes of medications across age groups, sex, degree of polypharmacy, and HRQoL categories. In this case, we dichotomized HRQoL (low PCS, high PCS, low MCS, and high MCS) using the rank procedure in SAS. |
|  |  | (*c*) If relevant, consider translating estimates of relative risk into absolute risk for a meaningful time period. |  |  |

Continued on next page

| Other analyses | 17 | Report other analyses done—eg analyses of subgroups and interactions, and sensitivity analyses | 9, 13 | Rao-Scott Chi-square test was applied to the categorical variables to determine significant differences in the population characteristics across degrees of polypharmacy.  Antihyperlipidemic and beta-adrenergic blocking agents were used across all groups (sex, age group, degree of polypharmacy, and HRQoL categories), and patients with low PCS and hyperpolypharmacy used a higher proportion of these two classes of medications. All groups used analgesics except for patients 65 years and older, and all groups used antidiabetic agents except those between 18 and 44 years and patients with minor polypharmacy. Diuretics, on the other hand, were used by males, patients with excessive polypharmacy, patients with low PCS and MCS, high MCS, and patients 65 years and older (Fig 2). Of the individuals taking analgesics drug class, acetaminophen-hydrocodone (28.01%) and tramadol (15.68%) were the number 1 and 2 medications taken by this subgroup. Additionally, among individuals taking diuretics, furosemide (60.07%) and hydrochlorothiazide (21.60%) were the number 1 and 2 medications in this class (Fig 3). Furthermore, for each therapeutic drug class, except for atorvastatin and simvastatin, within the antihyperlipidemic agent, the proportion of prescription medications not covered by insurance exceeded those covered by insurance (S1 Fig). |
| --- | --- | --- | --- | --- |
| Discussion | | | | |
| Key results | 18 | Summarise key results with reference to study objectives | 20 | The current study examined the association between polypharmacy and HRQoL among non-dialysis CKD patients. The results showed that polypharmacy is negatively associated with HRQoL. We found that major and hyperpolypharmacy were associated with lower PCS by 3 and 4 points compared to patients with minor polypharmacy. Our study also revealed that major and hyperpolypharmacy were associated with lower MCS by 0.37 and 1 point compared to patients with minor polypharmacy.  To the best of our knowledge, this is the first study to investigate the association of polypharmacy and HRQoL among non-dialysis CKD patients using nationally representative survey data in the United States. |
| Limitations | 19 | Discuss limitations of the study, taking into account sources of potential bias or imprecision. Discuss both direction and magnitude of any potential bias | 23 | However, there are limitations in our study that are worth noting. First, the design of this study is cross-sectional, thereby precluding the ability to infer causality. It remains indeterminate whether the association between low HRQoL and polypharmacy stems from the introduction of more medications, or an increase in comorbidities over time and vice versa. Second, it is possible that the HRQoL of CKD patients could be influenced by factors such as the severity of CKD or the addition of more comorbidities. However, the assessment of CKD severity was hindered since the MEPS data does not provide information on the different stages of CKD. Third, recall bias could result since MEPS is based on a self-report data collection process. The number of medications prescribed may not be accurately represented since it is likely that individuals may recall only the most recent medications prescribed. Fourth, since MEPS data only collects information on prescribed medications, our study did not include the number of medications resulting from over-the-counter use. Fifth, the glomerular filtration rate (GFR) is a standard indicator used in assessing the presence of CKD. However, given the self-reported nature of the MEPS data, laboratory results such as GFR were not evaluated. |
| Interpretation | 20 | Give a cautious overall interpretation of results considering objectives, limitations, multiplicity of analyses, results from similar studies, and other relevant evidence | 23 – 24 | In conclusion, our study showed that major polypharamcy and hyperpolypharmacy among non-dialysis CKD patients was significantly associated with lower physical and mental HRQoL compared to patients with minor polypharmacy. The negative association between polypharmacy and physical HRQoL was much stronger than the negative association between polypharmacy and mental HRQoL. However, we observed that depression, severe mental disability, and anxiety were highest among individuals with hyperpolypharmacy compared to patients with major and minor polypharmacy. Depression had the strongest association with HRQoL for both PCS and MCS. Our study emphasizes the need for further assessment of the combination of medications taken by non-dialysis CKD patients to reduce unnecessary and inappropriate drug combinations. Considering the impact of pill burden on the HRQoL among CKD patients and the possibility of some of these patients having hyperpolypharmacy, we recommend that healthcare providers be aware of this and review the medication list regularly for these patients. Additionally, a future prospective study is needed to further assess how pill burden affects the quality of life of non-dialysis CKD patients by evaluating both the positive and negative consequences of the prescribed medications. Future studies should also investigate the clinical implications of utilizing multiple medications beyond a certain threshold and their impact on the mental and physical health of non-dialysis CKD patients. |
| Generalisability | 21 | Discuss the generalisability (external validity) of the study results |  |  |
| Other information | |  | | |
| Funding | 22 | Give the source of funding and the role of the funders for the present study and, if applicable, for the original study on which the present article is based | 24 | The author(s) received no specific funding for this work. |

*Give information separately for cases and controls in case-control studies and, if applicable, for exposed and unexposed groups in cohort and cross-sectional studies.

**Note:** An Explanation and Elaboration article discusses each checklist item and gives methodological background and published examples of transparent reporting. The STROBE checklist is best used in conjunction with this article (freely available on the Web sites of PLoS Medicine at http://www.plosmedicine.org/, Annals of Internal Medicine at http://www.annals.org/, and Epidemiology at http://www.epidem.com/). Information on the STROBE Initiative is available at www.strobe-statement.org.
