# Supplementary figures and images for: The association between polypharmacy and health-related quality of life among non-dialysis chronic kidney disease patients
Source: PLoS One. 2023 Nov 13;18(11):e0293912. doi: 10.1371/journal.pone.0293912 (PMC10642842; doi:10.1371/journal.pone.0293912)

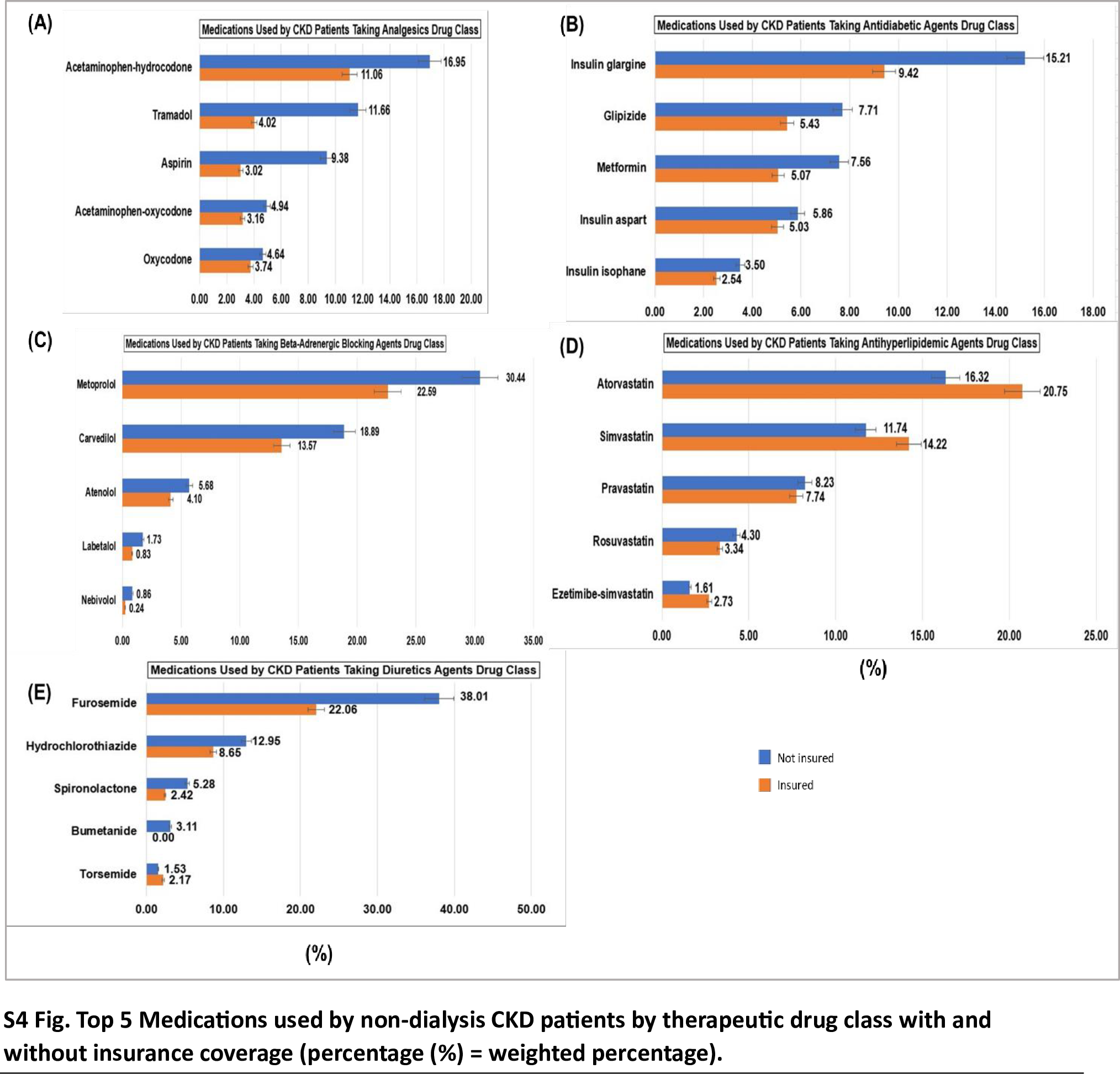

Supplement: S1 Fig — (TIF) [file pone.0293912.s002.tif]
