# Supplementary material for: The association between polypharmacy and health-related quality of life among non-dialysis chronic kidney disease patients
Source: PLoS One. 2023 Nov 13;18(11):e0293912. doi: 10.1371/journal.pone.0293912 (PMC10642842; doi:10.1371/journal.pone.0293912)
Supplement: Attachment — Submitted filename: Response to Reviewers.docx [file pone.0293912.s006.docx]

We extend our gratitude for the second time to the reviewers for generously dedicating their valuable time and expertise towards reviewing our manuscript. Their insightful review has made a significant contribution to improving the quality of this manuscript. We have combined all comments again and meticulously addressed each comment in accordance with the reviewers’ recommendations. The remarks from the reviewers have been formatted in italics for clarity. The changes can be seen in track changes in the file “Revised Manuscript with Track Changes.”

Reviewer #1

1. *In last review I questioned about the polypharmacy groupings. Authors say, “we classified polypharmacy based on the number of unique therapeutic classes of prescribed medications taken concomitantly over a specific round (e.g., 6-month data collection period) for each year”. According to study description, same person can be captured in data in multiple years. For a given person, the number of medications likely to fluctuate over time, therefore the person will be grouped into different polypharmacy groups in different years. This creates major implications on statistical analysis because chisquare test and OLS regression etc require each person be in a single polypharmacy group. How was this handled in the statistical analyses?*.
   - **Authors response**: Thank you for the opportunity to clarify this point. Each year the Agency for Healthcare Research and Quality (AHRQ) releases two sets of data: The consolidated data and a longitudinal data file. The consolidated yearly released data files are designed such that individuals that appeared in the current year data are independent from individuals that appear in the previous year data. Each participant is assigned unique weights and variance estimation strata. Excluding individuals from the data with the assumption that they appeared in the previous year will completely compromise the integrity of the data.
   - On the other hand, the released longitudinal datasets are such that an individuals can appear in two consecutive years. With such a scenario, AHRQ assigns similar weights and variance estimation strata indicating that persons appearing in the previous year and current year are similar.
   - For this study, we utilized the consolidated yearly released data files indicating that patients who appeared in the previous year's data files are independent of patients who appeared in the current year. We couldn't use the longitudinal data file because the number of individuals in the follow-up year was very small, leading to a much smaller sample size (Line number 118 to 121 and 170 to 172).
2. *In the last review I asked if the list of most commonly used drugs can be driven by the access issues (eg; cost, covered by insurance or public funds, etc) rather than by the medical issues. As a response authors say, “we have controlled for health insurance and prescription drug coverage in our analysis”. I am not willing to accept this explanation because statistical controlling is relevant when presenting something estimated from a (multivariable) statistical model, it is irrelevant when presenting a descriptive list as observed in data. However, I like to accept their next explanation that says “We believe the assessment of cost will be outside the scope of our current study”*.
   - **Authors response**: Thank you for calling our attention to this important additional information. As a supporting document in the manuscript, we have included a chart on insurance coverage by drug (S4 Fig). Also see chart below.

**S4 Fig. Top 5 Medications used by non-dialysis CKD patients by therapeutic drug class with and without insurance coverage (percentage (%) = weighted percentage).**

3. *The authors have taken the comments on board and have largely improved the manuscript. The limitations are now clearer. I still struggle with the definition of "excessive" polypharmacy, as the indications for the various medications were not known.*
   - **Authors response:** Thank you for pointing this out again. Another term that is also used in the literature is hyperpolypharmacy (see the listed articles below). All terms with excessive polypharmacy in the manuscript have been changed to hyperpolypharmacy.
   - References:

Masnoon N, Shakib S, Kalisch-Ellett L, Caughey GE. What is polypharmacy? A systematic review of definitions. BMC geriatrics. 2017 Dec;17:1-0.

Palmer K, Villani ER, Vetrano DL, Cherubini A, Cruz-Jentoft AJ, Curtin D, Denkinger M, Gutierrez-Valencia M, Guðmundsson A, Knol W, Mak DV. Association of polypharmacy and hyperpolypharmacy with frailty states: a systematic review and meta-analysis. European Geriatric Medicine. 2019 Feb 15;10:9-36.

Herrinton LJ, Lo K, Alavi M, Alexeeff SE, Butler KM, Chang C, Chang CC, Chu VL, Krishnaswami A, Deguzman LH, Prausnitz S. Effectiveness of Bundled Hyperpolypharmacy Deprescribing Compared With Usual Care Among Older Adults: A Randomized Clinical Trial. JAMA Network Open. 2023 Jul 3;6(7):e2322505-..

Kennel PJ, Kneifati-Hayek J, Bryan J, Mehta K, Banerjee S, Sobol I, Safford M, Goyal P. Prevalence and Determinants of Hyperpolypharmacy in Adults with Heart Failure. Journal of Cardiac Failure. 2018 Aug 1;24(8):S33.

4. *Also, I would consider a major conclusion to be that the* *pill burden is high among patients with a known diagnosis of CKD, and that given this it is possible that some of this polypharmacy may be "excessive", therefore clinicians should be aware and review the medication list regularly.* *Also a prospective study is required to assess the associated pill burden and deeper investigation into the contributors ot reduced quality of life may help to understand the good or bad effect of the prescriptions.*
   - **Authors response:** Thank you for this additional useful statement. It has been included in the manuscript in line number 402 to 406.

Reviewer #2

1. *The only outstanding issue to correct is that I see an error in the revision is response to my second point about medication under-prescribing (lines 327-332). This should refer to UNDER prescribing of RAAS inhibitors, Vit D and statins, and OVER prescribing of NSAIDs. At present, this appears the wrong way around.*
- **Authors response:** Thank you for pointing out this transcript error. We have corrected it in the manuscript (Line number 329 to 332).

We have included all datasets that we worked with at this point (analytical and medication dataset). We do not have any other dataset at our disposal. All datasets that we have worked with can be publicly assessed from the medical panel survey data website. <https://www.ahrq.gov/data/meps.html>

In the manuscript, the link to reference list #2 was changed from <https://dph.georgia.gov/sites/dph.georgia.gov/files/related_files/site_page/kidney_factsheet%20(1)%20cdc.pdf> to <https://dpacmi.org/documents/kidney_Factsheet.pdf> (The previous link can no longer be found). The same information was found in the new link.
